# Characteristics of drug‐related deaths among individuals identified as LGBTQ+ in the United Kingdom, 1997–2024

**DOI:** 10.1111/add.70198

**Published:** 2025-09-20

**Authors:** Emmert Roberts, Miriam Hillyard, Caroline Copeland

**Affiliations:** ^1^ National Addiction Centre, Institute of Psychiatry, Psychology and Neuroscience King's College London and the South London and the Maudsley NHS Foundation Trust London UK; ^2^ National Addiction Centre, Institute of Psychiatry, Psychology and Neuroscience King's College London London UK; ^3^ Faculty of Life Sciences and Medicine King's College London London UK

**Keywords:** chemsex, drug‐related deaths, epidemiology, gamma‐butyrolactone, LGBTQ+, sexualised drug use

## Abstract

**Background and Aims:**

Individuals from sexual and gender minorities (LGBTQ+) are understudied and at increased and differential risk of experiencing drug‐related harms when compared with the general population. We aimed to determine the case characteristics, circumstances of death and type of implicated drugs among LGBTQ+ individuals dying due to drug‐related causes. We also aimed to assess any differences between deaths occurring in the context of sexualised vs. non‐sexualised drug use.

**Design:**

Retrospective cohort study.

**Setting:**

Coronial records submitted to the National Programme on Substance Use Mortality (NPSUM) in the United Kingdom (UK), 1997–2024.

**Cases:**

Decedents identified as LGBTQ+.

**Measurements:**

Information was available on decedent sociodemographics, characteristics of death and drugs implicated in death.

**Findings:**

A total of 83 decedents were identified as LGBTQ+. Forty‐six were identified as cis men (55.4%), four as trans men (4.8%), two as cis women (2.4%) and 31 as trans women (37.3%). Forty‐five were identified as gay, including 44 gay men (53.0%) and one gay woman (1.2%), with three identified as bisexual (3.6%). Decedents were predominantly of White ethnicity (*n* = 68, 81.9%) with a mean age of 38.2 years (standard deviation 12.1; range 16–84). Overall, 16 (19.8%) cases were deemed intentional. Poisoning was the main disease or condition that was certified as the underlying cause of death (*n* = 46, 55.4%). The median number of drugs implicated in death was 2 [Interquartile range (IQR) 1, 2] with multiple drug toxicity implicated in the majority of cases (*n *= 42, 50.6%). The two most common drug groups implicated in death were opioids (*n* = 31, 37.3%) and gamma‐hydroxybutyrate (GHB) and related compounds (*n* = 14, 16.9%). Death occurred in the context of sexualised drug use in 21 cases (25.3%). There were statistically significantly fewer cases in which any opioid (33.9% vs. 4.8%, *P* = 0.009) or any benzodiazepine (21.0% vs. 0.0%, *P* = 0.02) were implicated when compared with cases of non‐sexualised drug use.

**Conclusions:**

Over the last three decades in the United Kingdom there have been consistent numbers of drug‐related deaths each year in which individuals were identified as LGBTQ+, results likely representing conservative estimates. A minority of drug‐related deaths occurred in the context of sexualised drug use.

## INTRODUCTION

In recent years multiple countries have reported their highest number of drug‐related deaths since records began. [1] International research consistently reports differential drug use patterns and an increased risk of drug‐related harms among individuals who identify as belonging to sexual and gender minorities [i.e. lesbian, gay, bisexual, trans, queer and others (LGBTQ+)] [2, 3].

Despite improved understanding of the drivers for increased prevalence and different patterns of drug use among LGBTQ+ individuals, [2] and a recent greater emphasis on understanding healthcare inequities in potentially vulnerable and marginalised groups, [1] there has been limited systematic epidemiological examination of drug‐related fatalities within this population. Few jurisdictions routinely collect or report drug‐related deaths by sexual orientation or trans status and, when they do, they tend to focus on specific substances or subpopulations that may not be representative of wider trends. [4] To our knowledge, only one previous national study has examined drug‐related deaths among LGBTQ+ individuals in the United Kingdom (UK), reporting on 21 cases before 2013 in which gamma‐hydroxybutyrate (GHB) and related compounds were implicated in death [5].

Substantial concerns have also been raised about the emergence of specific drug‐related harms, particularly among men who have sex with men (MSM), in the context of sexualised drug use or ‘chemsex’ [6]. Reports highlight that the preponderance of specific drug use profiles (typically stimulants and compounds related to GHB) and participant characteristics (e.g. a high prevalence of injecting drug use) may lead to differential risk of drug‐related death within these circumstances [7]. There has been similarly limited systematic exploration of drug‐related fatalities in this context with sparse data availability consistently highlighted as a barrier to developing understanding [6]. If specific case characteristics and/or particular drug use profiles were found to be associated with fatalities among LGBTQ+ individuals or occurring in the context of sexualised drug use within this community, this could further understanding and lead to the development of targeted interventions or specific harm reduction advice.

To address these gaps, we aimed to determine: (1) the case characteristics; (2) the circumstances of death; and (3) the number and type of drugs implicated in death among LGBTQ+ people dying because of drug‐related causes in the United Kingdom and assess any differences stratified by if death occurred in the context of sexualised drug use.

## METHODS

This study is reported according to the Strengthening the Reporting of Observational Studies in Epidemiology (STROBE) statement, [8] the completed checklist available as Table S1 in the online supplementary material.

### The National Programme on Substance Use Mortality

We conducted a retrospective cohort study using records drawn from the National Programme on Substance Use Mortality (NPSUM), this article containing recycled methods text material from previous NPSUM publications reported in accordance with guidance from the text recycling research project (https://textrecycling.org) [9]. This database contains information on an observational cohort that collects coronial data on deaths at any age related to any psychoactive drug, other than nicotine, caffeine or where the only substance implicated is alcohol. Alcohol is reported within NPSUM if death is related to any other psychoactive drug [9]. In total, NPSUM holds records on more than 55 000 deaths with reports received from England, Wales, the Channel Islands and the Isle of Man since 1997. Additional reports were received from the Scottish Crime and Drug Enforcement Agency between 2004 and 2011 and from the General Register Office for Northern Ireland since 2004. Deaths are referred to a coroner if the cause of death is unknown, is violent or unnatural, sudden and unexplained, occurred during or following a period of anaesthesia, or may have been caused by an industrial disease or poisoning. Coronial files typically include the coroner's decision as to cause of death, statements from witnesses, family and friends, first responders (e.g. police, emergency services), general practitioner (GP) records and hospital records. Toxicology is discretionally requested by individual coroners, and coronial jurisdictions choose to voluntarily report all deaths within their area to NPSUM if any of the following criteria are met: (1) psychoactive substance(s) are directly implicated in death; (2) an individual has a history of dependence or misuse of drugs; or (3) if controlled drugs are identified at post‐mortem. The King's College London (KCL) Biomedical and Health Sciences, Dentistry, Medicine and Natural and Mathematical Sciences Research Ethics Subcommittee (BDM RESC) re‐confirmed in August 2024 that NPSUM does not require Research Ethics Committee (REC) review as all individuals are deceased.

### Case identification

All cases reported between 1997 and November 2024 were identified and NPSUM fields searched for the following terms: gay*, lesbian*, trans*, queer*, *sex*, *gender* and *binary in addition to any cases in which it was reported that decedents were known by an alias. Authors manually reviewed the text of coronial files to confirm each decedent had been identified as LGBTQ+, with individuals identified as transgender if their coronial documentation reported that they were in the process of transitioning, had transitioned, were under the care of a gender service or had a documented diagnosis of gender dysphoria. These terms were selected in collaboration with coronial staff and people with LGBTQ+ identities. Final searches of NPSUM were conducted on the 24 June 2025.

### Measures

Data on decedent socio‐demographics, mental health history (i.e. a documented history of individual mental health conditions), substance use history (i.e. documented history of substance dependence and injecting status) and which individual substances were ultimately deemed implicated in death were extracted from coronial files. All cases additionally recorded the findings concerning the disease or condition that led directly to death, any antecedent cause(s) and other significant conditions contributory to death. Case files were manually screened to determine if coronial records indicated that death occurred in the context of sexualised drug use.

### Data and statistical analysis

We generated descriptive statistics for all decedents. Means, SDs and ranges were presented for continuous variables, as well as median and interquartile range (IQR) in skewed distributions. Overall percentages were reported for all binary and categorical variables and the valid percentage (i.e. the percentage of cases where medical or narrative history was available) was reported for mental health, substance use and sexualised drug use variables. Continuous variables were compared using an unpaired *t* test, ordinal variables using the Wilcoxon rank sum test and categorical variables using a χ^2^ test. Where cell sizes were five or less categorical variables were compared using Fisher's exact test [10, 11]. All analyses were conducted in STATA IC version 16 with the significance level set at 0.05. For reference, comparative data for the full NPSUM cohort can be found in the online supplementary material as Tables S2 and S3. The analysis plan was not pre‐registered and the results should be considered exploratory.

## RESULTS

A total of 83 decedents were identified as LGBTQ+ from coronial records at the time of their death following drug use in the United Kingdom between 1997 and 2024.

### Socio‐demographics and case characteristics

Socio‐demographic and case characteristics of all decedents can be found in Table 1.

**TABLE 1:** Socio‐demographic, case characteristics, circumstances of death and mental health and substance use history of people dying because of drug‐related causes identified as LGBTQ+ at the time of their death in the United Kingdom, 1997–2024.

		All (*n* = 83), *n* (%)	Sexualised drug use (*n* = 21), *n* (%)
Age	Mean (y)	38.2 (SD = 12.1; range = 16–84)	44.3 (SD = 6.3; range = 32–55)
Sex assigned at birth	Female	6 (7.2)	0 (0.0)
	Male	77 (92.8)	21 (100.0)
Ethnicity	White	68 (81.9)	17 (81.0)
	Black	2 (2.4)	0 (0.0)
	Indian	1 (1.2)	1 (4.8)
	Unknown/not recorded	12 (14.5)	3 (14.3)
Occupation^a^	Employed (manual)	14 (16.9)	6 (28.6)
	Employed (non‐manual)	20 (24.1)	7 (33.3)
	Unemployed	27 (32.5)	4 (19.0)
	Student	3 (3.6)	0 (0.0)
	Self‐employed	4 (4.8)	1 (4.8)
	Retired/invalid/sickness	3 (3.6)	0 (0.0)
	Unknown	12 (14.5)	3 (14.2)
Year of death	1997–2002	12 (14.5)	1 (4.8)
	2003–2007	19 (22.9)	5 (23.8)
	2008–2012	26 (31.3)	9 (42.9)
	2013–2017	10 (12.0)	1 (4.8)
	2018–2024	16 (19.3)	5 (23.8)
Place of death	Own place of residence	50 (60.2)	9 (42.9)
	Other residential address	6 (7.2)	3 (14.2)
	Hostel	2 (2.4)	0 (0.0)
	Hospital	13 (15.7)	4 (19.0)
	Sauna	3 (3.6)	3 (14.2)
	Other^b^	9 (10.8)	2 (9.5)
Direct cause of death (ICD‐10 code)	Poisoning, accidental (X40–X45)	32 (38.5)	8 (38.1)
	Poisoning, intentional (X60–X84)	7 (8.4)	0 (0.0)
	Poisoning, undetermined intent (T40.5, Y10–Y14)	7 (8.4)	1 (4.8)
	Other (e.g. T71, J18 *etc*.)	21 (25.3)	8 (34.7)
	Unascertained (R99)/not reported	16 (19.2)	4 (19.0)
Mental health^c^	People with a history of any mental health disorder	29 (37.2)	4 (19.0)
	People with a history of a depressive disorder	17 (21.8)	2 (9.5)
Addiction^c^	People with a history of substance dependence	41 (62.1)	7 (53.8)
	People with an injecting history	5 (19.2)	1 (20.0)
Sexual orientation	People identified as a gay man	44 (53.0)	18 (85.7)
	People identified as a gay woman	1 (1.2)	0 (0.0)
	People identified as bisexual	3 (3.7)	1 (4.7)
Trans status	People identified as transgender	35 (42.1)	**2 (9.5)**

*Note*: Variables in bold represent those that are significantly different (*P* < 0.05) between deaths occurring in the context of sexualised drug use compared to deaths occurring in the context of non‐sexualised drug use.

^a^ Occupation definitions are based on those used by the United Kingdom Office of National Statistics (ONS) [12].

^b^ Includes business addresses, open spaces/parks, streets/roads and public buildings/places.

^c^ Valid percentage reported.

Overall decedents were predominantly cisgender (*n* = 48, 57.8%) with 46 identified as cis men (55.4%), four as trans men (4.8%), two as cis women (2.4%) and 31 as trans women (37.3%). With respect to sexual orientation, 45 were identified as gay, including 44 gay men (53.0%) and one gay woman (1.2%), with three identified as bisexual (3.6%) and none identified as queer or non‐binary. Decedents were predominantly of White ethnicity (*n* = 68, 81.9%) and had a mean age of 38.2 years (SD = 12.1; range = 16–84). A substantial number were reported as employed in non‐manual professions at the time of their death (*n* = 20, 24.1%) and more than half of all decedents died while in their own accommodation (*n* = 50, 60.2%). All cases contained narrative information on the circumstances of death and in 21 (25.3%) information submitted to the coroner contained reports that death occurred in the context of sexualised drug use.

When compared to deaths that did not occur in the context of sexualised drug use, the only significant differences among people dying in the context of sexualised drug use were that decedents were significantly older [36.1 years (12.9) vs. 44.4 years (6.3), *P* = 0.006] and there were significantly fewer decedents identified as transgender (53.2% vs. 9.5%, *P* < 0.005).

### Characteristics of death and mental health and substance use history

Circumstances of death and mental health and substance use histories of all decedents can be found in Table 1. With respect to the manner of death, the majority of cases were deemed of accidental intent (*n* = 54, 66.7%), 16 were deemed intentional (19.8%) with the remainder deemed of undetermined intent (*n* = 12, 14.5%) or natural causes (*n* = 1, 1.2%). Poisoning was the main disease or condition that was certified as the direct cause of death with coronial determination reporting 46 cases directly because of poisoning (55.4%), of which 32 were deemed accidental [38.6%, International Classification of Diseases, 10th Revision (ICD‐10) codes, X40, X41, X42, X44 and X45], seven intentional (8.4%; ICD‐10, X61, X62 and X63) and seven undetermined (8.4%; ICD‐10, T40.5, Y11 and Y12). Of the remaining 37 (44.6%) deaths, the direct cause was unascertained in 16 cases (19.3%), with multiple other direct causes in the remaining 21 cases (25.3%) including asphyxiation (e.g. suffocation by a plastic bag) (*n* = 3, 3.6%) and respiratory pathology (e.g. bronchopneumonia) (*n* = 4, 4.8%).

In cases where medical history was available, approximately a third of all decedents had a history of any mental health condition (*n* = 29/78, 37.2%), the most common documented mental health condition being a depressive disorder (*n* = 17/78, 21.8%). Over half of all decedents had a history of substance dependence (*n* = 41/66, 62.1%), with almost a fifth of decedents having a history of injecting (*n* = 5/26, 19.2%). There were no significant differences among the cohort dying in the context of sexualised drug use.

### Number and type of drugs implicated in death

The number and type of drugs implicated in death can be found in Table 2. The median number of drugs implicated in death was 2 (IQR = 1, 2) with multiple drug toxicity (i.e. ≥2 drugs) implicated in the majority of cases (*n* = 42, 50.6%). The two most common drug groups implicated in death were opioids (*n* = 31, 37.3%) and GHB and related compounds (*n* = 14, 16.9%) with the three most common individual drugs implicated being heroin (*n* = 15, 18.1%), alcohol (*n* = 13, 15.7%) and cocaine (*n* = 12, 14.5%). Nearly half of decedents had a drug for which they had a prescription implicated in death (*n* = 40, 48.2%).

**TABLE 2:** The number and type of drugs implicated in death among people dying because of drug‐related causes identified as LGBTQ+ at the time of their death in the United Kingdom, 1997–2024.

Drug implicated in death^a^	All (*n* = 83), *n* (%)	Sexualised drug use (*n* = 21), *n* (%)	Non‐sexualised drug use (*n* = 62), *n* (%)
All			
Mean no. of drugs implicated	1.9 (SD = 1.1; range = 1–5)	1.7 (SD = 0.9; range = 1–4)	1.9 (SD = 1.1; range = 1–5)
Median no. of drugs implicated	2 (IQR = 1, 2; range = 1–5)	1 (IQR = 1, 2, range = 1–4)	2 (IQR = 1, 4; range = 1–5)
Only single substance implicated	41 (49.4)	12 (57.1)	28 (45.2)
Multiple substances implicated	42 (50.6)	9 (42.9)	34 (54.8)
Prescribed			
Any prescribed drug implicated	40 (48.2)	**6 (28.6)**	**34 (54.8)**
Opioids			
Any opioid	31 (37.3)	**1 (4.8)**	**21 (33.9)**
Heroin	15 (18.1)	1 (4.8)	14 (22.6)
Methadone	7 (8.4)	0 (0.0)	7 (11.3)
Benzodiazepines			
Any benzodiazepine	13 (15.7)	**0 (0.0)**	**13 (21.0)**
Diazepam	6 (7.2)	0 (0.0)	6 (9.7)
Antidepressants			
Any antidepressant	17 (20.5)	**1 (4.8)**	**16 (25.8)**
GHB and related compounds			
GHB/GBL^b^	14 (16.9)	6 (28.6)	8 (12.9)
Alcohol^c^	13 (15.7)	1 (4.8)	12 (19.4)
Cocaine	12 (14.5)	5 (23.8)	7 (11.3)
MDMA	9 (10.8)	3 (14.3)	6 (9.7)

*Note*: Variables in bold represent those that are significantly different (*P* < 0.05) between deaths occurring in the context of sexualised drug use compared to deaths occurring in the context of non‐sexualised drug use. Abbreviations: GBL, gamma‐butyrolactone; GHB, gamma‐hydroxybutyrate; IQR, interquartile range; LGBTQ+, lesbian, gay, bisexual, trans, queer and others; MDMA, 3,4‐methylenedioxymethamphetamine; NPSUM, National Programme on Substance Use Mortality.

^a^ Only those substances implicated in five or more deaths are reported.

^b^ Including GBL, which is rapidly metabolised by the body into GHB.

^c^ The nature of the NPSUM database is such that deaths in which alcohol is the only substance implicated are not recorded.

When compared to deaths that did not occur in the context of sexualised drug use, there was a significant difference (*P* < 0.001) in the overall drug profile implicated in death among people dying in the context of sexualised drug use. When differences in each implicated drug group or individual drug were examined *post hoc* the only significant differences were significantly fewer decedents had any prescribed drug implicated (54.8% vs. 28.6%, *P* = 0.04), any opioid implicated (33.9% vs. 4.8%, *P* = 0.009), any benzodiazepine implicated (21.0% vs. 0.0%, *P* = 0.02) or any antidepressant implicated (25.8% vs. 4.8%, *P* = 0.04).

Stratified results for gay men and trans women are available in the online supplementary material as Tables S4 and S5.

## DISCUSSION

Over the last three decades within the NPSUM cohort it was possible to identify low, but consistent numbers of deaths each year following drug use among LGBTQ+ individuals. While the case characteristics are in some ways typical of the wider UK NPSUM drug‐related deaths over the same timeframe, including a high proportion of ethnically White decedents and people dying in their own residence, atypically there were substantially higher numbers of deaths because of intentional poisoning and higher rates of employment, particularly in non‐manual professions [9, 12]. With respect to the drugs implicated in death, there were substantially reduced numbers of deaths in which any opioid was implicated, with GHB and MDMA overrepresented. Significantly fewer deaths of people identified as trans occurred in the context of sexualised drug use along with significantly fewer deaths in which any opioid, benzodiazepine or antidepressant was implicated.

The preponderance of cocaine, GHB and 3,4‐methylenedioxymethamphetamine (MDMA) use is similar to drug‐use profiles seen in other Western samples of LGBTQ+ individuals, however the low proportion of deaths implicating opioid use, particularly within a sexualised context, is notable and consistent with previously observed drug use profiles. [4] Previous research has consistently demonstrated that LGBTQ+ individuals who use drugs may face significant and unique barriers to access evidence‐based addiction treatment and harm reduction services with documented reasons including compounded stigma, lack of access to ‘culturally competent’ services that are able to address their needs, and limited treatment offers for problematic use of substances that may be more prevalent in LGBTQ+ populations (e.g. GHB or MDMA) [13]. These results suggest that the current typical UK treatment service orientation toward treatment of problematic alcohol or opioid use may not be viewed as meeting the needs of LGBTQ+ individuals given the observed profile of drugs implicated in fatalities [14]. Given the high number of people in employment observed within this LGBTQ+ population, restrictive, only within working hours, service operating hours may also represent a barrier for this group. It should be noted that a minority of deaths occurred in the context of sexualised drug use, including a significantly lower number among people identified as trans. As such, practitioners and policymakers need to be aware that drug‐related death in LGBTQ+ populations is not restricted to ‘chemsex’ contexts and, while sexualised drug use pathways remain vital, it should not be assumed that all LGBTQ+ individuals who use drugs require ‘chemsex’‐related interventions [6]. A number of previous studies have highlighted the increased risk of suicidal behaviours within LGBTQ+ populations, and this study adds to that literature, demonstrating high numbers of drug‐related deaths where the direct cause is certified as intentional self‐poisoning [15]. Ensuring adequate exploration of mental illness and suicidality during drug service and wider healthcare assessments is warranted. Previous work has highlighted the utility of including LGBTQ+ people with lived and living experience of drug use in treatment policy and service design, with improved trust, access and outcomes demonstrated for these groups when meaningful involvement has been facilitated [16].

The study has several strengths including the granular examination of substances implicated in death and the large time frame. There are, however, several important limitations. Coroners do not routinely record sexual orientation or trans status, and therefore, to identify LGBTQ+ individuals and death occurring in the context of sexualised drug use, the study relies on documentation submitted to the coroner to both contain this information, and for it to be accurate [17]. There are likely a substantial number of individuals within NPSUM where this may have either not been established by those submitting coronial evidence, or where their LGBTQ+ status or the sexualised circumstances of their death were not deemed pertinent to report. As such the numbers reported in this study are likely to represent significantly conservative estimates, particularly given that the 2021 UK census estimates the general population prevalence of LGB+ individuals as 3.2% and trans people as 0.5% (www.ons.gov.uk/census). This low number of cases may also result in limited power to examine potential differences among subgroups (e.g. deaths occurring in the context of sexualised drug use). The lack of a significant difference in some specific implicated drugs (e.g. GHB) is potentially reflective of insufficient power to detect a difference within this context. Nevertheless, to our knowledge, this is the largest epidemiological study to examine drug‐related deaths within this population and the socio‐demographic, case characteristics and implicated drug profiles have material utility to provide information on this under‐researched group. A further limitation within NPSUM is that potential variables of interest may either not be available or their quality and completion inconsistent across coronial jurisdictions. There are significant proportions of incomplete reporting for some variables (e.g. over a sixth of cases contain no reported ethnicity), which limits the ability to comment on key factors that may expose significant inequities. Because of the voluntary nature of NPSUM submissions, it has incomplete coverage of the United Kingdom coronial system over the studied timeframe, with variability of toxicological examination in all cases, therefore, some drug‐related deaths will likely have been missed, and some implicated substances not identified [9].

In summary, it was possible to identify consistent numbers of drug‐related deaths where decedents were LGBTQ+ individuals over the past three decades in the United Kingdom, despite non‐routine reporting of sexual orientation or trans status in coronial records. Given the unique profile of drug‐related deaths within this population, we would argue for routine monitoring and reporting of sexual orientation and trans status within official death registration statistics and other databases monitoring drug‐related harms, to further understand and monitor these trends. Meaningful inclusion in treatment policy and service design should be a priority for this group, given the substantial barriers to treatment often faced by these individuals.

## AUTHOR CONTRIBUTIONS

**Emmert Roberts:** Conceptualization (equal); formal analysis (lead); funding acquisition (lead); investigation (lead); methodology (lead); project administration (lead); resources (equal); writing—original draft (lead). **Miriam Hillyard:** Conceptualization (equal); investigation (equal); validation (equal); writing—review and editing (equal). **Caroline Copeland:** Conceptualization (equal); data curation (lead); investigation (equal); project administration (equal); resources (lead); supervision (lead); validation (equal); writing—review and editing (equal).

## DECLARATION OF INTERESTS

All authors have completed the ICJME Unified Competing Interest form (available on request from the corresponding author) and declare: no support from any organisation for the submitted work; no financial relationships with organisations that might have an interest in the submitted work in the previous 3 years, no other relationships or activities that could appear to have influenced the submitted work.

## Supporting information


**Table S1.** Strengthening the Reporting of Observational Studies in Epidemiology (STROBE) statement checklist [1].
**Table S2.** Sociodemographic, case characteristics, circumstances of death and mental health and substance use history of all people within the NPSUM database dying due to drug‐related causes in the United Kingdom, 1997–2024.
**Table S3.** The number and type of drugs implicated in death among all people within the NPSUM database dying due to drug‐related causes in the United Kingdom, 1997–2024.
**Table S4.** Sociodemographic, case characteristics, circumstances of death and mental health and substance use history among gay men and trans women dying due to drug‐related causes in the United Kingdom, 1997–2024.
**Table S5.** The number and type of drugs implicated in death among gay men and trans women dying due to drug‐related causes in the United Kingdom, 1997–2024.

## Data Availability

Data are available subject to institutional approvals.
