# Supplementary material for: Characteristics of drug‐related deaths among individuals identified as LGBTQ+ in the United Kingdom, 1997–2024
Source: Addiction. 2025 Sep 20;121(1):208–14. doi: 10.1111/add.70198 (PMC12710844; doi:10.1111/add.70198)
Supplement: Supplementary file 1 — Table S1. Strengthening the Reporting of Observational Studies in Epidemiology (STROBE) statement checklist [1]. Table S2. Sociodemographic, case characteristics, circumstances of death and mental health and substance use history of all people within the NPSUM database dying due to drug‐related causes in the United Kingdom, 1997–2024. Table S3. The number and type of drugs implicated in death among all people within the NPSUM database dying due to drug‐related causes in the United Kingdom, 1997–2024. Table S4. Sociodemographic, case characteristics, circumstances of death and mental health and substance use history among gay men and trans women dying due to drug‐related causes in the United Kingdom, 1997–2024. Table S5. The number and type of drugs implicated in death among gay men and trans women dying due to drug‐related causes in the United Kingdom, 1997–2024. [file ADD-121-208-s001.docx]

**Online Supplementary Material**

Contents

Tables

S1. Strengthening the Reporting of Observational Studies in Epidemiology (STROBE) statement checklist (1)

S2. Sociodemographic, case characteristics, circumstances of death and mental health and substance use history of all people within the NPSUM database dying due to drug-related causes in the United Kingdom, 1997 - 2024

S3. The number and type of drugs implicated in death among all people within the NPSUM database dying due to drug-related causes in the United Kingdom, 1997 - 2024

S4. Sociodemographic, case characteristics, circumstances of death and mental health and substance use history among gay men and trans women dying due to drug-related causes in the United Kingdom, 1997 - 2024

S5. The number and type of drugs implicated in death among gay men and trans women dying due to drug-related causes in the United Kingdom, 1997 - 2024

Tables

Table S1: Strengthening the Reporting of Observational Studies in Epidemiology (STROBE) statement checklist (1)

|  | Item No | Recommendation |
| --- | --- | --- |
| **Title and abstract** | 1 | (*a*) Indicate the study’s design with a commonly used term in the title or the abstract; Title: Page 1 |
|  |  | (*b*) Provide in the abstract an informative and balanced summary of what was done and what was found; Abstract: Page 3 |
| Introduction | | |
| Background/rationale | 2 | Explain the scientific background and rationale for the investigation being reported; Introduction: Page 5 |
| Objectives | 3 | State specific objectives, including any prespecified hypotheses; Introduction: Page 5/6 |
| Methods | | |
| Study design | 4 | Present key elements of study design early in the paper; Methods: Page 7/8 |
| Setting | 5 | Describe the setting, locations, and relevant dates, including periods of recruitment, exposure, follow-up, and data collection; Methods: Pages 7/8 |
| Participants | 6 | (*a*) Give the eligibility criteria, and the sources and methods of selection of participants. Describe methods of follow-up; Methods: Pages 7/8 |
|  |  | (*b*) For matched studies, give matching criteria and number of exposed and unexposed N/A |
| Variables | 7 | Clearly define all outcomes, exposures, predictors, potential confounders, and effect modifiers. Give diagnostic criteria, if applicable Methods: Page 7/8 |
| Data sources/ measurement | 8* | For each variable of interest, give sources of data and details of methods of assessment (measurement). Describe comparability of assessment methods if there is more than one group Methods: Page 7/8 |
| Bias | 9 | Describe any efforts to address potential sources of bias Methods: Page 7/8 |
| Study size | 10 | Explain how the study size was arrived at Methods Page 7/8; Results Page 10 |
| Quantitative variables | 11 | Explain how quantitative variables were handled in the analyses. If applicable, describe which groupings were chosen and why Methods Page 9 |
| Statistical methods | 12 | (*a*) Describe all statistical methods, including those used to control for confounding Methods Page 9 |
|  |  | (*b*) Describe any methods used to examine subgroups and interactions N/A |
|  |  | (*c*) Explain how missing data were addressed Methods Page 9 |
|  |  | (*d*) If applicable, explain how loss to follow-up was addressed N/A |
|  |  | (*e*) Describe any sensitivity analyses N/A |
| Results | | |
| Participants | 13* | (a) Report numbers of individuals at each stage of study—eg numbers potentially eligible, examined for eligibility, confirmed eligible, included in the study, completing follow-up, and analysed Results Page 10 |
|  |  | (b) Give reasons for non-participation at each stage N/A |
|  |  | (c) Consider use of a flow diagram N/A |
| Descriptive data | 14* | (a) Give characteristics of study participants (eg demographic, clinical, social) and information on exposures and potential confounders Results Pages 10/11 |
|  |  | (b) Indicate number of participants with missing data for each variable of interest Table 1 |
|  |  | (c) Summarise follow-up time (eg, average and total amount) Methods Page 10/11 |
| Outcome data | 15* | Report numbers of outcome events or summary measures over time Table 1 |
| Main results | 16 | (*a*) Give unadjusted estimates and, if applicable, confounder-adjusted estimates and their precision (eg, 95% confidence interval). Make clear which confounders were adjusted for and why they were included Table 1 |
|  |  | (*b*) Report category boundaries when continuous variables were categorized Table 1 |
|  |  | (*c*) If relevant, consider translating estimates of relative risk into absolute risk for a meaningful time period N/A |
| Other analyses | 17 | Report other analyses done—eg analyses of subgroups and interactions, and sensitivity analyses Table 1/2 Results Pages 10/11 |
| Discussion | | |
| Key results | 18 | Summarise key results with reference to study objectives Discussion Page 13 |
| Limitations | 19 | Discuss limitations of the study, taking into account sources of potential bias or imprecision. Discuss both direction and magnitude of any potential bias Discussion Page 14 |
| Interpretation | 20 | Give a cautious overall interpretation of results considering objectives, limitations, multiplicity of analyses, results from similar studies, and other relevant evidence Discussion Page 13/14 |
| Generalisability | 21 | Discuss the generalisability (external validity) of the study results Discussion Page 13 |
| Other information | | |
| Funding | 22 | Give the source of funding and the role of the funders for the present study and, if applicable, for the original study on which the present article is based Title Page 2 |

1. Von Elm E, Altman DG, Egger M, Pocock SJ, Gøtzsche PC, Vandenbroucke JP. The Strengthening the Reporting of Observational Studies in Epidemiology (STROBE) statement: guidelines for reporting observational studies. Annals of internal medicine. 2007;147(8):573-7.

Table S2: Sociodemographic, case characteristics, circumstances of death and mental health and substance use history of all people within the NPSUM database dying due to drug-related causes in the United Kingdom, 1997 - 2024

|  | All, n=55,522  % | |  | All, n=55,522  % | |
| --- | --- | --- | --- | --- | --- |
| **Age** | Mean (Years) | 40.7 (SD 13.4; Range 0 - 105) | **Place of death** | Own place of residence | 55.1 |
| **Sex Assigned at Birth** | Female | 26.8 |  | Other residential address | 15.5 |
|  | Male | 73.2 |  | Hostel | 0.9 |
| **Ethnicity** | White | 65.4 |  | Hospital | 17.3 |
|  | Black | 1.0 |  | Sauna | <0.01 |
|  | Asian | 0.6 |  | Other^2^ | 16.2 |
|  | Unknown/Not Recorded | 32.0 | **Mental health^3^** | People with a history of any mental health disorder | 25.1 |
| **Occupation^1^** | Employed (Manual) | 16.4 |  | People with a history of a depressive disorder | 16.8 |
|  | Employed (Non-Manual) | 16.7 | **Addiction^3^** | People with a history of substance dependence | 72.6 |
|  | Unemployed | 43.7 |  | People with an injecting history | 17.2 |
|  | Student | 1.7 | **Sexual orientation** | People identified as a gay man | <0.01 |
|  | Self-employed | 1.4 |  | People identified as a gay woman | <0.01 |
|  | Retired/Invalid/Sickness | 9.0 |  | People identified as bisexual | <0.01 |
|  | Unknown | 18.5 | **Trans status** | People identified as transgender | <0.01 |
| **Year of Death** | 1997 - 2002 | 15.5 |  |  |  |
|  | 2003 - 2007 | 18.2 |  |  |  |
|  | 2008 - 2012 | 19.2 |  |  |  |
|  | 2013 - 2017 | 16.6 |  |  |  |
|  | 2018 - 2024 | 30.6 |  |  |  |

^SD Standard Deviation; 1 Occupation definitions are based on those used by the United Kingdom Office of National Statistics (ONS) (18)^

^2 Includes business addresses, open spaces/parks & public buildings/places; 3 Percentage of all NPSUM cases from 2010 - Nov 1st 2024, as opposed to the valid percent where past medical history is available^

Table S3: The number and type of drugs implicated in death among all people within the NPSUM database dying due to drug-related causes in the United Kingdom, 1997 - 2024

| Drug implicated in death | | All, n=55,522  n (%) |
| --- | --- | --- |
| All | Mean number of drugs implicated | 2.1 (SD 1.5; Range 0 - 14) |
|  | Median number of drugs implicated | 2 |
|  | Only single substance implicated^1^ | 36.1 |
|  | Multiple substances implicated^1^ | 57.9 |
| Opioids | Any opioid | 67.2 |
|  | Heroin | 41.4 |
|  | Methadone | 18.9 |
| Benzodiazepines | Any benzodiazepine | 19.1 |
|  | Diazepam | 14.7 |
| Antidepressants | Any antidepressant | 17.0 |
| Gamma-hydroxybutyrate (GHB) and related compounds | GHB/GBL^2^ | 0.5 |
| Alcohol | Alcohol^3^ | 23.2 |
| Cocaine | Cocaine | 16.0 |
| 3,4-methylenedioxymethamphetamine (MDMA) | MDMA | 2.1 |

^SD Standard Deviation;^

^1 Percentage does not add up to 100% as there are some cases with zero implicated substances e.g., where a chronic drug user has died of^

^sepsis so is concluded at inquest as having suffered a drug-related death, but it is their long-term drug use that has resulted in death as opposed^

^to the toxicity of individual drugs^

^2 Gamma-butyrolactone (GBL) is rapidly metabolised by the body into gamma-hydroxybutyrate (GHB)^

^3 The nature of the NPSUM database is such that deaths in which alcohol is the only substance implicated are not recorded.^

S4. Sociodemographic, case characteristics, circumstances of death and mental health and substance use history among gay men and trans women dying due to drug-related causes in the United Kingdom, 1997 - 2024

|  | | Gay Men  (n=44) n (%) | Trans women  (n=31) n (%) |  | | Gay Men  (n=44) n (%) | Trans women (n=31) n (%) |
| --- | --- | --- | --- | --- | --- | --- | --- |
| **Age** | Mean (Years) | 39.7 (SD 9.6; Range 16 - 60) | 36.0 (SD 15.0; Range 18 - 84) | **Place of death** | Own place of residence | 26 (59.1) | 19 (61.3) |
| **Sex Assigned at Birth** | Female | 0 (0.0) | 0 (0.0) |  | Other residential address | 4 (9.1) | 2 (6.5) |
|  | Male | 44 (100.0) | 31 (100.0) |  | Hostel | 0 (0.0) | 2 (6.5) |
| **Ethnicity** | White | 41 (93.2) | 20 (64.5) |  | Hospital | 8 (18.2) | 2 (6.5) |
|  | Black | 0 (0.0) | 2 (6.5) |  | Sauna | 3 (6.8) | 0 (0.0) |
|  | Indian | 1 (2.3) | 0 (0.0) |  | Other^2^ | 3 (6.8) | 6 (19.4) |
|  | Unknown/Not Recorded | 2 (4.6) | 9 (29.0) | **Direct cause of death**  **(ICD-10 code)** | Poisoning, accidental (X40-X45) | 24 (54.5) | 6 (19.4) |
| **Occupation^1^** | Employed (Manual) | 11 (25.0) | 3 (9.7) |  | Poisoning, intentional (X60-X84) | 4 (9.1) | 3 (9.7) |
|  | Employed (Non-manual) | 16 (36.4) | 2 (6.5) |  | Poisoning, undetermined intent (T40.5, Y10-Y14) | 3 (6.8) | 4 (12.9) |
|  | Unemployed | 8 (18.2) | 18 (58.0) |  | Other (e.g., T71, J18 etc.) | 8 (18.2) | 8 (25.8) |
|  | Student | 1 (2.3) | 1 (3.2) |  | Unascertained (R99) / Not reported | 5 (11.4) | 10 (32.2) |
|  | Self-employed | 2 (4.6) | 1 (3.2) | **Mental health^3^** | People with a history of any mental health disorder | 11 (25.6) | 13 (48.1) |
|  | Retired/Invalid/Sickness | 1 (2.3) | 2 (6.5) |  | People with a history of a depressive disorder | 7 (16.3) | 6 (19.4) |
|  | Unknown | 5 (11.4) | 4 (12.9) | **Addiction^3^** | People with a history of substance dependence | 20 (60.6) | 14 (53.8) |
| **Year of Death** | 1997 - 2002 | 7 (15.9) | 3 (9.7) |  | People with an injecting history | 2 (13.3) | 3 (27.3) |
|  | 2003 - 2007 | 14 (31.8) | 3 (9.7) |  |  |  |  |
|  | 2008 - 2012 | 16 (36.4) | 9 (29.0) |  |  |  |  |
|  | 2013 - 2017 | 1 (2.3) | 9 (29.0) |  |  |  |  |
|  | 2018 - 2024 | 6 (13.6) | 7 (22.6) |  |  |  |  |

^SD Standard Deviation; 1 Occupation definitions are based on those used by the United Kingdom Office of National Statistics (ONS) (16)^

^2 Includes business addresses, open spaces/parks, streets/roads and public buildings/places; 3 Valid percentage reported^

S5. The number and type of drugs implicated in death among gay men and trans women dying due to drug-related causes in the United Kingdom, 1997 - 2024

| Drug implicated in death^1^ | | Gay men  (n=44) n (%) | Trans women  (n=31) n (%) |
| --- | --- | --- | --- |
| All | Mean number of drugs implicated | 1.9 (SD 1.1; Range 1 - 5) | 1.7 (SD 1.0; Range 1 - 4) |
|  | Median number of drugs implicated | 2 (IQR 1,3; Range 1 - 5) | 1 (IQR 1,2; Range 1 - 4) |
|  | Only single substance implicated | 19 (43.2) | 14 (45.2) |
|  | Multiple substances implicated | 25 (56.8) | 17 (54.8) |
| Prescribed | Any prescribed drug implicated | 16 (36.4) | 13 (41.9) |
| Opioids | Any opioid | 11 (25.0) | 16 (51.6) |
|  | Heroin | 3 (6.8) | 10 (32.3) |
|  | Methadone | 3 (6.8) | 1 (3.2) |
| Benzodiazepines | Any benzodiazepine | 4 (9.1) | 6 (19.4) |
|  | Diazepam | 1 (2.3) | 2 (6.5) |
| Antidepressants | Any antidepressant | 7 (15.9) | 9 (29.0) |
| Gamma-hydroxybutyrate (GHB)  and related compounds | GHB/GBL^2^ | 13 (30.0) | 1 (3.2) |
| Alcohol | Alcohol^3^ | 9 (20.5) | 3 (9.7) |
| Cocaine | Cocaine | 9 (20.5) | 3 (9.7) |
| 3,4-methylenedioxymethamphetamine (MDMA) | MDMA | 7 (15.9) | 1 (3.2) |

^1 Only those substances implicated in five or more deaths are reported^

^2 Including Gamma-butyrolactone (GBL) which is rapidly metabolised by the body into gamma-hydroxybutyrate (GHB)^

^3 The nature of the NPSUM database is such that deaths in which alcohol is the only substance implicated are not recorded.^
